# Iguratimod suppresses Tfh cell differentiation in primary Sjögren’s syndrome patients through inhibiting Akt/mTOR/STAT3 signaling

**DOI:** 10.1186/s13075-023-03109-4

**Published:** 2023-08-22

**Authors:** Taibiao Lyu, Hui Jiang, Liuting Zeng, Suying Liu, Chengmei He, Chaowen Luo, Lin Qiao, Yan Zhao, Hua Chen

**Affiliations:** 1 Department of Rheumatology and Clinical Immunology, Peking Union Medical College Hospital, Chinese Academy of Medical Sciences & Peking Union Medical College, 1 Shuaifuyuan, Beijing, 100730 China; 2 Key Laboratory of Rheumatology and Clinical Immunology, Ministry of Education, Beijing, China; 3 National Center of Dermatologic and Autoimmune Diseases, Beijing, China

**Keywords:** Primary Sjögren’s syndrome, Iguratimod, Follicular helper T cell

## Abstract

**Background:**

Iguratimod (IGU) reduces hypergammaglobulinemia and disease activity in pSS (primary Sjögren’s syndrome) patients. However, the therapeutical mechanism of IGU for pSS remains largely unknown. This study aimed to investigate the regulation of Tfh cell differentiation by IGU in pSS patients.

**Methods:**

We prospectively enrolled 13 pSS patients treated with IGU for 3 months and examined circulating T cell and B cell subsets by flow cytometry. We measured Tfh cell differentiation treated by IGU in pSS patients and healthy controls. Transcriptome analysis combined with molecular docking were employed to identify potential therapeutical targets of IGU, which were verified by Western blot and Tfh cell differentiation.

**Results:**

Tfh, plasmablast, and plasma cells were suppressed by IGU treatment at 1 and 3 months. Tfh cell differentiation and function were significant inhibited by IGU in pSS patients and healthy controls in vitro. Pyruvate dehydrogenase kinase 1 (PDK1) was identified as a target of IGU during Tfh cell differentiation, and the downstream Akt phosphorylation was attenuated by IGU. Moreover, the activity of mTORC1 and phosphorylation of STAT3 were suppressed by IGU, with downregulation of *BCL6* and upregulation of *PRDM1.* Finally, Akt activator restored IGU-suppressed Tfh cell differentiation.

**Conclusions:**

IGU suppresses Tfh cell differentiation in pSS patients through interacting with PDK1 and suppressing Akt-mTOR-STAT3 signaling.

**Supplementary Information:**

The online version contains supplementary material available at 10.1186/s13075-023-03109-4.

## Background

Primary Sjögren’s syndrome (pSS) is a chronic systemic autoimmune disease featured with salivary, lacrimal, and other exocrine gland manifestations, with a high prevalence of 0.5–1.5% globally [1, 2]. Besides xerophthalmia and xerostomia, about half of pSS patients present hematologic, musculoskeletal, pulmonary, neurologic, and other systemic manifestations, which increase disease burden and mortality despite aggressive glucocorticoid and immunosuppressant therapy [3].

B cell overactivation is a hallmark of pSS, including polyclonal hypergammaglobulinemia composing rheumatoid factor (RF), antinuclear autoantibodies, and other autoantibodies, which are essential players for systemic manifestations [4]. B cell-targeted therapies are tested in pSS [5]. Ianalumab, an anti-BAFF receptor antibody, significantly improves disease activity and stimulates salivary flow and decreases peripheral B cells and immunoglobins [6, 7]. Telitacicept, a TACI-Ig targeting both BAFF and APRIL, dramatically improves disease activity and reduces immunoglobins and B cells in phase 2 trial [8]. Remibrutinib, a BTK inhibitor, shows an improvement in disease activity in phase 2 study [9]. Rituximab improves fatigue and serum immunoglobins [5]. These encouraging data suggest that targeting B cells is a promising therapeutical approach for pSS.

Iguratimod (IGU) is a new DMARD approved for rheumatoid arthritis (RA) in China and Japan, with anti-inflammatory and immunomodulatory properties [10]. We and others have reported that IGU significantly reduced immunoglobin and disease activity in pSS patients, which is a promising agent for pSS. IGU significantly decreases total B cells, BAFF-receptor^+^ B cells, activated B cells, and plasma cells in pSS patients [11, 12], suggesting IGU suppresses B cell differentiation. However, the therapeutical mechanism of IGU for pSS remains largely unknown.

Follicular helper T (Tfh) cells are a subset of CD4^+^ T cells expressing C-X-C chemokine receptor type 5 (CXCR5), programmed death-1 (PD-1), inducible T cell costimulator (ICOS)and B cell lymphoma 6 (*BCL6*), and downregulated PR domain zinc finger protein 1 (*PRDM1*). Tfh cells provide help to B cell activation, differentiation, and antibody affinity maturation in germinal center [13, 14] through CD40L [15], IL-21 [16, 17], and IL-4 [18]. Circulating CD4^+^CXCR5^+^ T (cTfh) cells share functional characteristics with Tfh cells [19]. Circulating and salivary gland Tfh cells are increased in pSS patients [20–22], positively correlated with serum immunoglobin and disease activity index [23, 24], which play a key role in pSS pathogenesis.

In light of IGU suppressing B cell differentiation and Tfh cells regulating B cell differentiation, we hypothesized that IGU potentially suppressed B cell differentiation through repressing Tfh cells. In this study, we examined the Tfh cells in pSS patients before and after IGU treatment, and gained insights into the underlying mechanism using transcriptome analysis.

## Methods

### Subjects

We prospectively enrolled 13 pSS patients who fulfilled the classification criteria of the 2002 American European Consensus Group from Peking Union Medical College Hospital (PUMCH) (Supplementary Table S1). Patients received 25 mg twice daily IGU due to clinical manifestations and did not receive glucocorticoids and immunosuppressants. Clinical features, laboratory tests, and circulating Tfh, Th1, Th2, Th17, switched memory B, plasmablasts, and plasma cells were assessed at baseline, month 1, and month 3. The study was approved by the institutional review board of PUMCH (No. HS-1112). All participants provided written informed consent.

### T cell isolation and differentiation

Peripheral blood mononuclear cells (PBMCs) were isolated from whole blood of pSS patients and HCs using Ficoll density gradient centrifugation. CD4^+^CD45RA^+^ naive CD4^+^ T cells were sorted from PBMCs with Naive CD4^+^ T Cell Isolation Kit II (Miltenyi Biotec) according to the manufacturer’s instructions, with a purity of over 95%. Naïve CD4^+^ T cells were activated with anti-CD3 (5 µg/ml, BD Bioscience) and anti-CD28 (5 µg/ml, BD Bioscience). TGF-β1 (5 ng/ml, R&D systems) plus IL-12 (1 ng/ml, R&D systems), anti-IL4 (10 µg/ml, PeproTech) plus IL-12 (10 ng/ml), IL-4 (2 ng/ml, PeproTech) plus anti-IFN-γ (10 µg/ml, BD Bioscience), and anti-IL4 (10 µg/ml) plus anti-IFN-γ (10 µg/ml) plus TGF-β (5 ng/ml, R&D systems) plus IL-1β (12.5 ng/ml, PeproTech) plus IL-6 (25 ng/ml, PeproTech) plus IL-23 (25 ng/ml, PeproTech), were supplemented for Tfh, Th1, Th2, and Th17 differentiation, respectively. T cells were treated with IGU (10 or 30 µg/ml, Simcere) or DMSO and were incubated in RPMI-1640 medium (Thermo Fisher) supplemented with 10% fetal bovine serum (FBS, Gibco), penicillin (100 U/ml), and streptomycin (100 U/ml) at 37 °C, 5% CO_2_ for 3–5 days. For T cell-B cell coculture, Tfh cells treated with IGU or DMSO under 5-day Tfh condition were washed in PBS and then were incubated with CD19^+^ B cells purified from PBMCs using CD19^+^ B cell Isolation Kit II (Miltenyi), supplemented with anti-CD3 (5 μg/ml), anti-CD28 (5 μg/ml), and CpG (2.5ug/mL, InvivoGen). B cells were collected at day 6 for flow cytometry.

### T cell proliferation, activation, and apoptosis assays

Naïve CD4^+^ T cells were labeled with 2 µM CFSE (BD Biosciences) for 15 min at 37 ℃, stimulated with anti-CD3 and anti-CD28, and were measured for proliferation using flow cytometry on day 3. T cell activation was assessed by staining with anti-CD69 at 16 h and anti-CD25 on day 3, and T cell apoptosis was examined on day 3 staining with Annexin-V and 7-AAD (BD Biosciences) for 15 min at room temperature.

### Flow cytometry

The following fluorochrome-conjugated antibodies purchased from Biolegend were used: CD4 (OKT4), CXCR5 (J252D4), PD-1 (A17188B), ICOS (C398.4A), IL-21, CD19 (HIB19), CD38 (S17015F), CD138(DL-101), IgD (IA6-2), CD27(M-T271), IFNγ (4S.B3), IL-4 (MP4-25D2), IL-17A (BL168), CD69 (FN50), and CD25 (BC96). Cells were incubated with antibodies at 4 °C at dark for 30 min for surface staining. For detecting IL-21, IL-17A, IL-4, and IFN-γ, cells were stimulated with PMA, ionomycin, and Brefeldin A for 6 h, then fixed and permeabilized using Cytofix/Cytoperm (BD Bioscience) 30 min at 4 °C, and were incubated with indicated antibodies for 1 h at 4 °C. Cells were analyzed with a BD Aria II Flow Cytometer (BD Bioscience), and data were processed using FlowJo X (Tree Star).

### Western Blot

Proteins were extracted using RIPA buffer (Solarbio, China) supplemented with protease inhibitors (Huaxingbio, China) and phosphatase inhibitors (Huaxingbio, China), quantified with a BCA Protein Assay kit (Aoqing Biotechnology, China) were subjected to 10% sodium dodecyl sulfate–polyacrylamide gel electrophoresis, transferred to polyvinylidene fluoride membrane (Millipore), blocked with QuickBlock Western Blocking Buffer (Beyotime), incubated with anti-p-Akt (Thr308, 1:1000, rabbit, Cell Signaling Technology), anti-pan-Akt (1:1000, rabbit, Cell Signaling Technology), anti-p-S6 (Ser235/236, 1:2000, rabbit, Cell Signaling Technology), anti-pan-S6 (1:1000, rabbit, Cell Signaling Technology), anti-p-STAT3(Tyr705, 1:1000, rabbit, Cell Signaling Technology), anti-pan-STAT3(1:1000, rabbit, Cell Signaling Technology), or anti-β-actin(1:2000, mouse, Easybio) at 4 °C overnight, and incubated with HRP-conjugated antibody for 1 h at room temperature. Bands were detected by Amersham Imager 680 and were analyzed using Image J (National Institutes of Health).

### Quantitative RT-PCR

Total RNA was extracted using TRIzol reagent (Thermo Fisher), was quantified by NanoDrop2000c spectrophotometer (NanoDrop Technologies), and was reverse transcribed into complementary DNA (cDNA) using PrimeScript RT Master Mix (Takara). Real-time PCR was performed using TB Green Premix Ex Taq II (Takara) and a Roche 480 II thermocycler, and relative expression against GAPDH was calculated using the comparative ΔΔCT method. The primer sequences are listed in Supplementary Table S2.

### Transcriptional analysis of IGU-treated CD4^+^ T cells

Total RNA was collected from naive CD4^+^ T cells cultured under Tfh condition and was sequenced by Novogene (China). After RNA qualification and quantification, the library was prepared with quality control. Then, sequencing was initiated, followed by read mapping (hg19) and gene expression quantification. Differential expression was calculated with DESeq2 v.1.26.0. *P*-values were adjusted according to Benjamini and Hochberg’s model. Differentially expressed genes were filtered based on adjusted *p-*value < 0.05 and **|**log2foldchange**|> **1. ClusterProfiler v.3.14.3 was used for Gene Ontology (GO) and Kyoto Encyclopedia of Genes and Genomes (KEGG) analysis.

### Target prediction and molecular docking

The molecular structure of IGU was obtained from PubChem and was searched in PharmMapper [25]. The potential targets of IGU were sorted for duplicates and were translated into gene names. Discovery Studio Client v.4.5 was used to hydrogenate proteins, remove water, and remove ligand molecules. AutoDock v.4.2 was used to convert compound molecules and protein molecules into “pdbqt” format and finally run Vina for molecular docking. If binding energy was less than 0, the compound (ligand) and protein (receptor) bind spontaneously. A binding energy ≤  − 5.0 kcal/mol was considered as ligand bind to receptor stably.

### Statistical analysis

Continuous variables were described as mean (standard deviation, SD) for normal distribution or median (interquartile range, IQR) for non-normal distribution, categorical variables were summarized as number (percentage). Comparisons between two groups were assessed using Student’s *t*-test or Wilcoxon-Mann–Whitney test for continuous variables and Fisher’s exact test for categorical variables as appropriate. Comparisons among three or more groups were assessed using analysis of variance (ANOVA) with Bonferroni adjusted *p*-value. A two-sided *p*-value < 0.05 was considered statistically significant. Data were analyzed using SPSS v.26.0 (IBM).

## Results

### IGU suppresses Tfh, plasmablast, and plasma cells in pSS patients

**Fig. 1:**
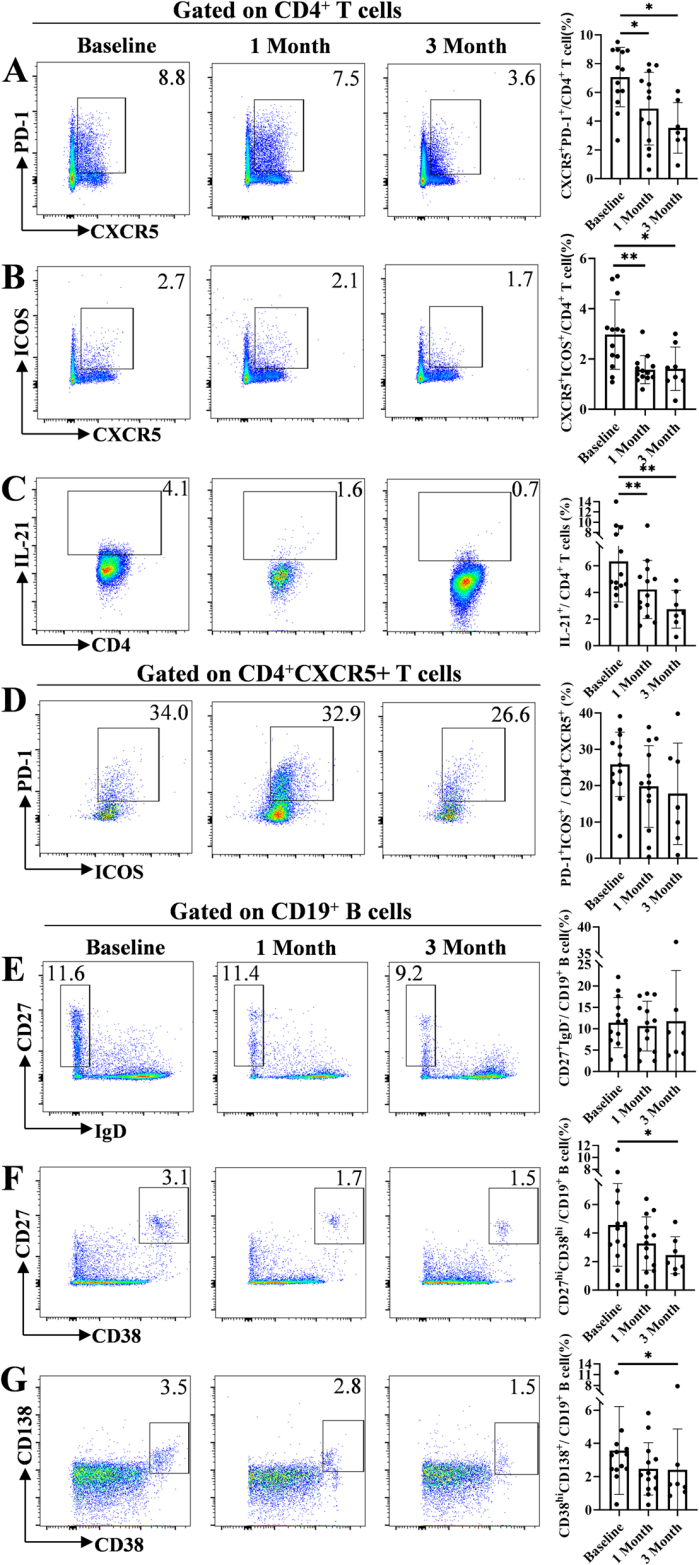
IGU suppresses Tfh cells in pSS patients. Flowcytometry analysis of (**A**) CD4^+^CXCR5^+^PD-1^+^, (**B**) CD4^+^CXCR5^+^ICOS^+^, (**C**) CD4^+^IL-21^+^, (**D**) CD4^+^CXCR5^+^PD-1^+^ICOS^+^ follicular T helper (Tfh) cells, (**E**) CD27^+^IgD^−^switched memory B cells, (**F**) CD38^hi^CD27^hi^ plasmablasts, and (**G**) CD38^hi^CD138^+^ plasma cells isolated from peripheral blood of pSS patients (*n* = 13). Data were presented as mean ± SD. Data were obtained from independent experiments. *p < 0.05, **p < 0.01 by ANOVA

To investigate the underlying mechanism of IGU-suppressed immunoglobin, we first examined the effector T cell subsets as well as B cell subsets in 13 IGU-treated patients (Supplementary Table S1) up to 3 months (Supplementary Figure S1). IGU treatment significantly decreased CXCR5^+^PD-1^+^, CXCR5^+^ICOS^+^, and IL-21^+^ Tfh cells (Fig. 1A–C) but not CXCR5^+^PD-1^+^ICOS^+^ Tfh cells (Fig. 1D). In contrast, IFN-γ^+^ Th1 cells and IL-4^+^ Th2 cells remained stable after IGU treatment (Supplementary Figure S2A and B), and IL-17A^+^ Th17 cells slightly decreased at 1 month (Supplementary Figure S2C). Furthermore, IGU treatment also suppressed CD27^hi^CD38^hi^ plasmablasts and CD38^hi^CD138^+^plasma cells (Fig. 1F and G) and reduced ESSDAI (Supplementary Figure S3A), which were correlated with Tfh cells (Supplementary Figure S4A-D). IGU also reduced IgG levels (Supplementary Figure S3B) but not lymphocyte counts (Supplementary Figure S3C) or CD27^+^IgD^−^ switched memory B cells (Fig. 1E). Together, these data indicated IGU treatment suppressed Tfh cells in pSS patient, which potentially played a role in attenuated B cell hyperactivation.

### IGU inhibits pSS Tfh cell differentiation in vitro

**Fig. 2:**
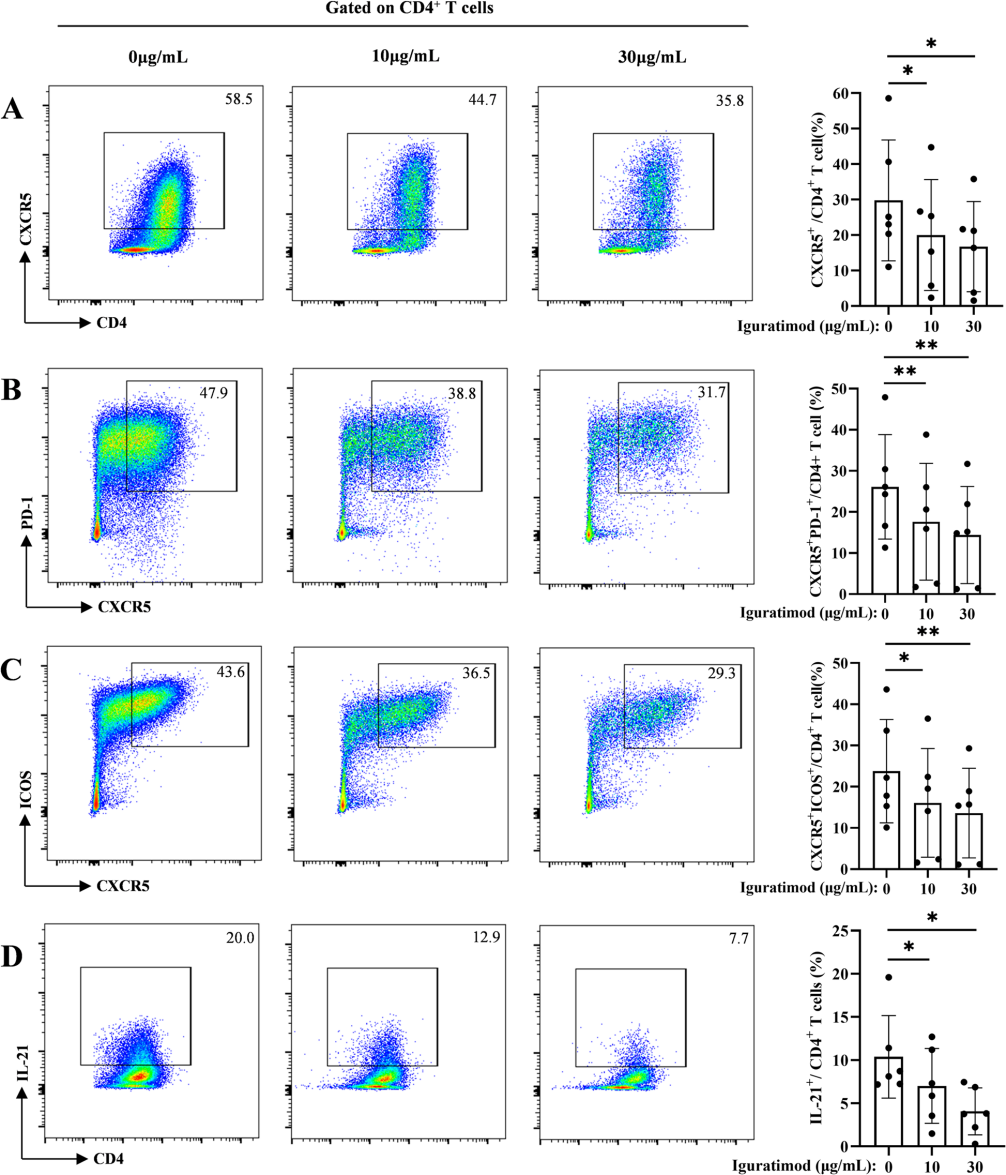
IGU attenuates pSS Tfh cell differentiation in vitro. Flowcytometry analysis of (**A**) CD4^+^CXCR5^+^, (**B**) CD4^+^CXCR5^+^PD-1^+^, (**C**) CD4^+^CXCR5^+^ICOS^+^, and (**D**) CD4^+^IL-21^+^ Tfh cells differentiated from pSS naive CD4^+^ T cells (*n* = 6) stimulated under Tfh condition for 5 days. Data were presented as mean ± SD. Data were obtained from three independent experiments. *p < 0.05, **p < 0.01 by ANOVA

Given Tfh cells regulate B cell maturation and IGU suppressed Tfh cells in pSS patients, we focused on IGU regulation on Tfh cells (Supplementary Figure S5). IGU suppressed T cell activation and proliferation, but not apoptosis (Supplementary Figure S6A-D). Consistently, IGU inhibited CXCR5^+^, CXCR5^+^PD-1^+^, CXCR5^+^ICOS^+^, and IL-21^+^ Tfh, and IL-17A^+^ Th17 cell differentiation (Supplementary Figure S7C) from both pSS (Fig. 2A–D) and healthy control (Supplementary Figure S8A-D) naive T cells in vitro and inhibited IFN-γ^+^ Th1 cells and IL-4^+^ Th2 cells differentiation at high concentration (Supplementary Figure S7A and B). Additionally, IGU suppressed CD27^+^IgD^−^ switched memory B cell differentiation (Supplementary Figure S9A), and IGU-treated Tfh cells attenuated B cell differentiation, including CD27^+^IgD^−^ switched memory B cells, CD27^hi^CD38^hi^ plasmablasts, and CD38^hi^CD138^+^ plasma cells (Supplementary Figure S10A-C). Therefore, these data suggested that IGU inhibited the activation, proliferation, and differentiation of Tfh cells, which further impaired the B cell differentiation.

### Transcriptome analysis of IGU-treated CD4^+^ T cells

**Fig. 3:**
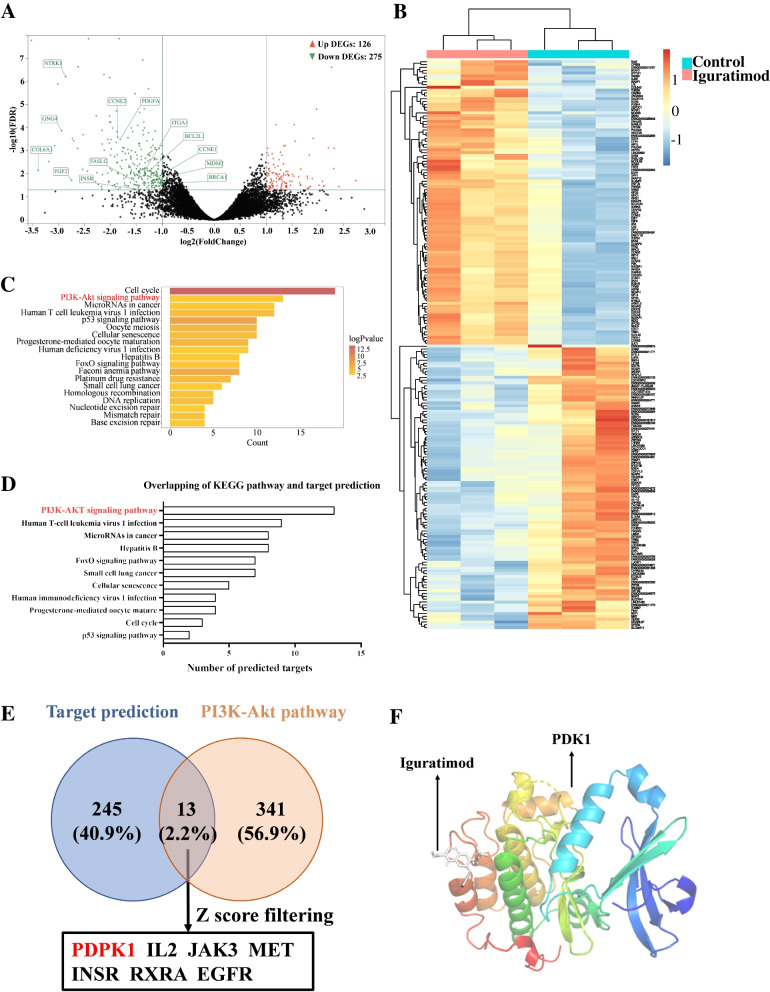
Transcriptome analysis of IGU-treated CD4^+^ T cells. **A** Volcano plot of DEGs between IGU-treated (*n* = 3) and untreated (*n* = 3) naïve CD4^+^ T cells under Tfh condition (red, upregulated, *n* = 126; green, downregulated, *n* = 275). **B** Heatmap of top 200 DEGs (up = 100, down = 100). **C** KEGG pathway enrichment analysis of DEGs. **D** Overlapping between KEGG pathway enrichment analysis and target prediction of IGU. **E** Venn diagram of predicted IGU targets and PI3K-Akt pathway. **F** Molecular docking diagram of binding sites of IGU on PDK1

We then conducted RNA-sequencing to elucidate the mechanism of IGU-inhibiting Tfh differentiation. IGU downregulated 275 genes and upregulated 126 genes (Fig. 3A) in CD4^+^ T cells, suggesting lower activation of T cells (Supplementary Figure S6A and B). The top 200 DEGs (up:100, down:100) showed distinct expression profiles of IGU-treated T cells (Fig. 3B). GO enrichment analysis revealed tubulin binding, chromosome segregation, and spindle were top enriched entities in molecular function, biological process, and cellular component, respectively (Supplementary Figure S11A-C). KEGG pathway enrichment analysis indicated 19 pathways were regulated by IGU, including the 2nd-ranked PI3K-Akt signaling pathway (Fig. 3C). We used target prediction based on pharmacophore mapping approach [25] to identify potential IGU-binding targets (Supplementary Table S3) and intersected with genes of enriched KEGG pathways (Supplementary Table S4, Supplementary Table S5). We identified that PI3K-Akt signaling pathway was the pathway with the most overlapping hits (Fig. 3D, Supplementary Table S6), including PDPK1, IL-2, JAK3, MET, INSR, RXRA, and EGFR. PDPK1, also known as pyruvate dehydrogenase kinase 1 (PDK1), was the top candidate of IGU binding according to *Z* score (Fig. 3E, Supplementary Table S6), which is a kinase phosphating AKT serine/threonine kinase 1 (Akt) in PI3K-Atk pathway. Finally, molecular docking validated the binding of IGU with PDK1 (Fig. 3F). Thus, transcriptome analysis implied that IGU regulated PI3K-Akt pathway through interaction with PDK1, which subsequently inhibited Tfh cell differentiation.

### IGU inhibits PDK1 and attenuates Akt-mTOR-STAT3 signaling

**Fig. 4:**
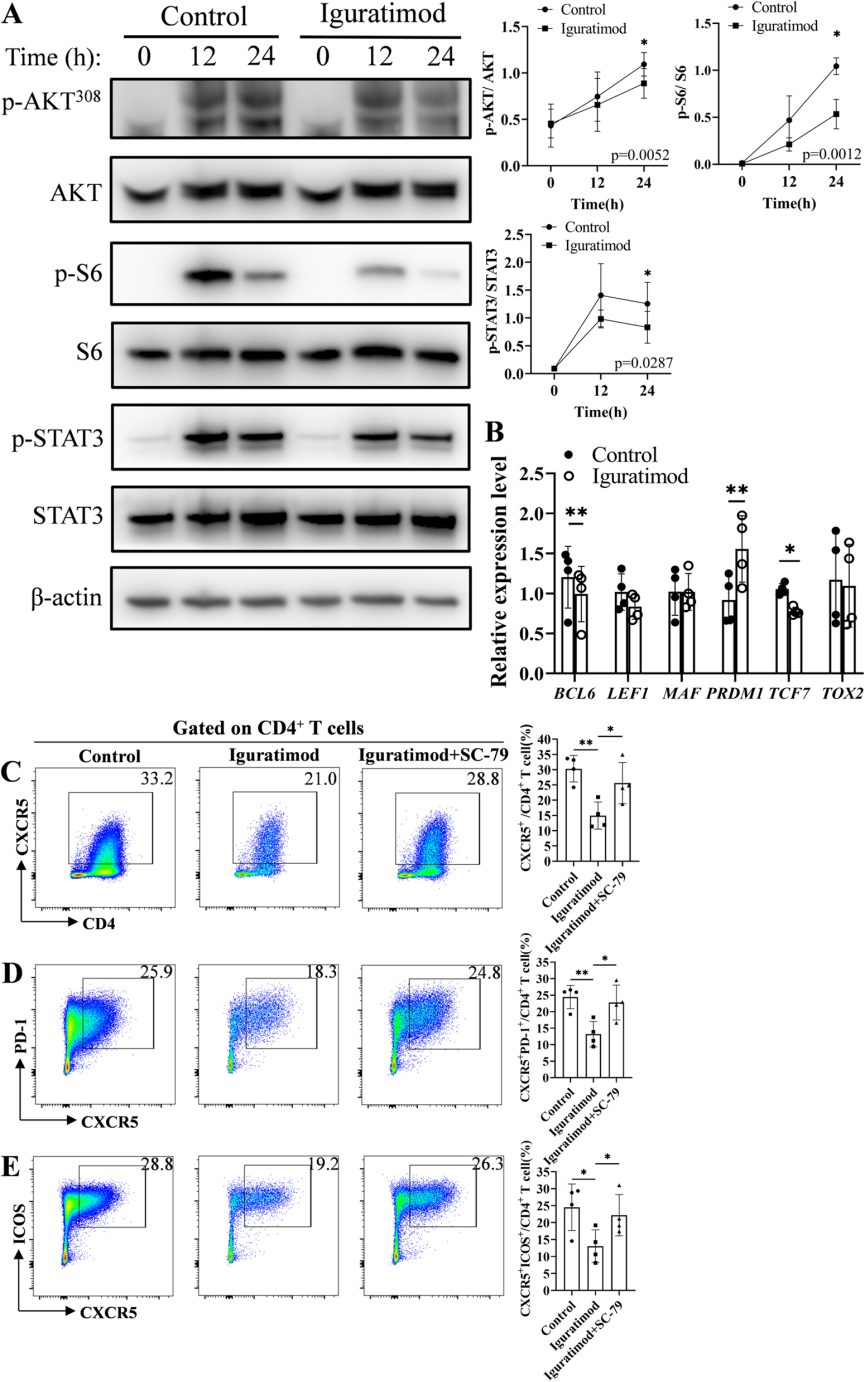
IGU inhibits Akt-mTORC1-STAT3 signaling by targeting PDK1. **A** Western blot analysis of Akt, mTORC1, and STAT3 in IGU-treated (*n* = 4) and untreated (*n* = 4) naïve CD4^+^ T cells incubated under Tfh condition for 0, 12, or 24 h. **B** Real-time qPCR analysis of Tfh-related transcription factors in IGU-treated (*n* = 4) and untreated (*n* = 4) Tfh cells. Flowcytometry analysis of **C** CD4^+^CXCR5^+^, **D** CD4^+^CXCR5^+^PD-1^+^, and **E** CD4^+^CXCR5^+^ICOS^+^ Tfh cells differentiated from DMSO-, IGU-, or IGU and SC-79-treated pSS naive CD4^+^ T cells (*n* = 4) under Tfh condition for 5 days. Data were presented as mean ± SD. Data were obtained from 2 independent experiments. *p < 0.05, **p < 0.01, ***p < 0.001 by paired Student’s *t*-test or ANOVA

Since Akt is phosphorylated by PDK1 and activates mechanistic/mammalian target of rapamycin complex 1 (mTORC1), which phosphorylates signal transducer and activator of transcription 3 (STAT3) [26], we next investigated whether Akt phosphorylation was regulated by IGU. AKT was phosphorylated during Tfh cell differentiation, which was significantly downregulated by IGU, indicating IGU inhibited AKT phosphorylation catalyzed by PDK1 (Fig. 4A). Furthermore, phosphorylation of S6, the substrate of mTORC1, and STAT3 were also downregulated in IGU-treated T cells, suggesting IGU inhibited Akt-mTOR-STAT3 signaling. Moreover, STAT3 regulated *BCL6* and *PRDM1*, the master transcription activator and transcription suppressor of Tfh, was downregulated and upgraded, respectively (Fig. 4B). To further validate IGU inhibited Tfh cell differentiation via Akt phosphorylation through PDK1, we used SC-79, a selective Akt activator, to rescue Akt phosphorylation in IGU-treated T cells. As expected, SC-79 restored S6 and STAT3 phosphorylation, T cell proliferation (Supplementary Figure S12), and reversed the decreased CXCR5^+^, CXCR5^+^PD-1^+^, CXCR5^+^ICOS^+^ Tfh cells in IGU-treated T cells (Fig. 4C–E). These data suggested that IGU inhibited PDK1-induced Akt phosphorylation, which suppressed mTORC1 activity and subsequent phosphorylation of STAT3, resulting in downregulation *BCL6* expression and upregulation *PRDM1*, and ultimately suppressed Tfh cell differentiation (Supplementary Figure S13).

## Discussion

In this study, we observed that IGU repressed Tfh cells as well as plasmablasts and plasma cells in pSS patients. Furthermore, we confirmed that IGU suppressed Tfh cell differentiation and B cell-helping function in vitro. Using transcriptome analysis combining molecular docking, we identified that IGU regulated PDK1 to inhibit PI3K-Akt signaling. Finally, we demonstrated that Akt-mTOR-STAT3 signaling was suppressed by IGU, resulting in downregulated *BCL6* and upregulated *PRMD1*, and ultimately attenuated Tfh cell differentiation.

B cell hyperactivation in pSS is significantly suppressed by IGU, which is partially induced by attenuated activated B cells, plasma cell differentiation, and BAFF signaling. In RA patients, IGU attenuates B cell terminal differentiation through downregulating PKC signaling and transcription factor EGR1 [27]. Tfh cells are essential for B cell activation, differentiation to plasma cells, antibody producing, antibody affinity maturation, and antibody-class switch [13], which plays pivotal role in autoantibody production of pSS, RA and lupus. In this study, we observed the IGU reduced Tfh cells, PD-1, ICOS, and IL-21 expressions in pSS patients, which are key molecules for B cell affinity maturation. Consistently, IGU treatment reduces serum IL-21 level in RA patients [28]. In vitro, RA Tfh cells in PBMCs treated by IGU shows an impaired secretion of IL-21 [29]. Thus, we proposed Tfh cells suppression as a novel mechanism of IGU treatment in pSS. IGU suppresses both B cell activation/differentiation and Tfh cell differentiation, which orchestrate to enhance B cell inhibition. Therefore, IGU is a promising immunomodulator for B cell overactivation and potential therapy targeting both B cells and Tfh cells in pSS. Given IGU suppresses Tfh cells in pSS and RA, IGU potentially suppresses Tfh cells in other Tfh-related autoimmune disease such as lupus [30, 31].

The mechanism of Tfh regulation by IGU is not fully understood. IGU inhibits RA T cell glycolysis via suppressing Hif1α-HK2 signaling [29]. We used transcriptome analysis to gain insights of signaling pathways regulated by IGU and combined analysis of potential IGU targets using molecular docking. Transcriptome analysis revealed that IGU suppressed PI3K-Akt signaling, but not NF-κB signaling, which is reported in synovial fibroblast-like cells [32]. Furthermore, PDK1, a key kinase of PI3K-Akt signaling, was suggested to interact with IGU. PI3K-Akt signaling is an essential pathway for T cell response [26, 33, 34], which favors Tfh and Th17 differentiation [35, 36]. PI3K promotes PDK1 relocation to phospholipid-enriched membrane for phosphorylation and activated PDK1 phosphates Akt to transduct signaling [37] (Supplementary Figure S13). Since PDK1 promoted Tfh cell differentiation via AKT phosphorylation, mTORC1 activation, and STAT3 phosphorylation [26], we validated that IGU indeed inhibited PI3K-Akt signaling and downstream mTORC1 and STAT3 activation in Tfh cells, which was reversed by Akt activator. IGU also downregulated *BCL6* and upregulated *PRDM1*, which are key transcription factors regulated by STAT3 [38] and synergically regulate Tfh genes including CXCR5, PD-1, and ICOS [39]. Our data highlighted a new Akt-mTOR-STAT3 signaling regulated by IGU in Tfh differentiation and added a new layer of therapeutically mechanism of IGU on T cells.

Ours study have limitations. We observed IGU effect on Tfh cells in pSS patients up to 3 months, and the long-term effect remains unclear. We observed a stable reduction in serum immunoglobin in small number of pSS patients treated with IGU for up to 12 months (data not show), and we are following the enrolled patient for 6 months and beyond to determine the long-term effect of IGU on Tfh cells and B cells. Our study strongly suggested an interaction between IGU and PDK1; however, the direct binding domain of PDK1 by IGU and whether IGU interfered the PDK1 activation remain unclear. A selected mutation of potential PDK1 binding sites analysis might yield more informative insights of this molecular mechanism.

## Conclusions

In summary, our study found that IGU suppresses Tfh cell differentiation in addition to B cell activation and differentiation. Mechanistically, IGU inhibits Akt-mTOR-STAT3 signaling by interacting with PDK1, subsequently represses *BCL6* and upregulates *PRDM1*, and ultimately inhibits Tfh differentiation. Our results shed new light on that IGU modulates both Tfh cells and B cells in pSS and provide mechanical evidence on the therapeutical effect of IGU in pSS.

### Supplementary Information

**Additional file 1: Supplementary Table S1.** Demographic characteristics of pSS cohort.

**Additional file 2: Supplementary Table S2.** PCR primers used in this study.

**Additional file 3: Supplementary Table S3.** Target prediction of iguratimod.

**Additional file 4: Supplementary Table S4.** Overlapping of enriched pathways gene set and target prediction analysis.

**Additional file 5: Supplementary Table S5.** KEGG pathway gene sets.

**Additional file 6: Supplementary Table S6.** Overlapping of PI3K-Akt pathway and target prediction analysis.

**Additional file 7: Supplementary Figure S1.** Gating strategy of peripheral T cells and B cells.

**Additional file 8: Supplementary Figure S2.** Peripheral T cell subsets in IGU-treated pSS patients.

**Additional file 9: Supplementary Figure S3.** Clinical and laboratory improvement in IGU-treated pSS patients.

**Additional file 10: Supplementary Figure S4.** Correlation analysis of Tfh cells with B cells and ESSDAI in IGU-treated pSS patients.

**Additional file 11: Supplementary Figure S5.** Gating strategy of T cell activation and differentiation.

**Additional file 12: Supplementary Figure S6.** IGU inhibits T cell activation and proliferation.

**Additional file 13: Supplementary Figure S7.** IGU inhibits Th1, Th2 and Th17 cell differentiation.

**Additional file 14: Supplementary Figure S8.** IGU inhibits healthy control Tfh cell differentiation.

**Additional file 15: Supplementary Figure S9.** IGU suppresses B cell differentiation.

**Additional file 16: Supplementary Figure S10.** IGU suppresses Tfh cell-facilitated B cell differentiation.

**Additional file 17: Supplementary Figure S11.** Gene ontology analysis of IGU-treated CD4^+^ T cells.

**Additional file 18: Supplementary Figure S12.** AKT activator restored IGU-suppressed mTOR and STAT3 phosphorylation and T cell proliferation

**Additional file 19: Supplementary Figure S13.** Diagram of mechanism of IGU-inhibited Tfh cell differentiation

## Data Availability

The original contributions presented in the study are included in the article/supplementary materials. Further inquiries can be directed to the corresponding authors.

## Abbreviations

Akt: Akt serine/threonine kinase 1
*Bcl6*: B cell lymphoma 6
CXCR5: C-X-C chemokine receptor type 5
Tfh: Follicular helper T
IGU: Iguratimod
ICOS: Inducible T cell costimulator
mTORC1: Mammalian target of rapamycin complex 1
*PRDM1*: PR domain zinc finger protein 1
pSS: Primary Sjögren’s syndrome
PD-1: Programmed death-1
PDK1: Pyruvate dehydrogenase kinase 1
RA: Rheumatoid arthritis
RF: Rheumatoid factor
STAT3: Signal transducer and activator of transcription 3
