# Supplementary material for: Iguratimod suppresses Tfh cell differentiation in primary Sjögren’s syndrome patients through inhibiting Akt/mTOR/STAT3 signaling
Source: Arthritis Res Ther. 2023 Aug 22;25:152. doi: 10.1186/s13075-023-03109-4 (PMC10463648; doi:10.1186/s13075-023-03109-4)
Supplement: Supplementary file 1 — Additional file 1: Supplementary Table S1. Demographic characteristics of pSS cohort. [file 13075_2023_3109_MOESM1_ESM.docx]

**Supplementary table S1.** Demographic characteristics of pSS cohort.

| **Variable** | **pSS patients (n=13)** |  |
| --- | --- | --- |
| Age (years), mean (±SD) | 44 (12) |  |
| Female sex, n (%) | 13 (100%) |  |
| Clinical features |  |  |
| Dry mouth, n (%) | 12 (92%) |  |
| Dry eyes, n (%) | 12 (92%) |  |
| Xerophthalmia, n (%) | 5 (38%) |  |
| Parotid swollen, n (%) | 3 (23%) |  |
| Positive LSG biopsy (n =4) | 4/4 |  |
| Extra-glandular involvements |  |  |
| Cutaneous, n (%) | 5 (38%) |  |
| Leukopenia, n (%) | 2 (15%) |  |
| ILD, n (%) | 1 (8%) |  |
| Laboratory features |  |  |
| IgG (g/L), mean (±SD) | 25 (7) | |
| RF (IU/mL), mean (±SD) | 176 (161) |  |
| ESR (mm/h), mean (±SD) | 34 (18) |  |
| ESSDAI, mean (±SD) | 5 (3) | |
| ESSPRI, mean (±SD) | 2 (1) |  |
| ANA positive, n (%) | 13 (100%) | |
| Anti-SSA positive, n (%) | 12 (92%) | |
| Anti-SSB positive, n (%) | 7 (54%) | |
| Treatment |  | |
| Hydroxychloroquine, n (%) | 10 (77%) | |

Quantitative data were expressed as mean with standard deviation (SD) for normal distribution. Categorical variables were expressed as counts and percentages. ANA, antinuclear antibody; Anti-SSA, Anti-Ro/Sjögren's syndrome A antigen; Anti-SSB, Anti-La/Sjögren's syndrome B antigen (SSB); ESR, erythrocyte sedimentation rate; IgG, Immunoglobulin G; ILD, interstitial lung disease; RF, rheumatoid factor.
