# Supplementary material for: Iguratimod suppresses Tfh cell differentiation in primary Sjögren’s syndrome patients through inhibiting Akt/mTOR/STAT3 signaling
Source: Arthritis Res Ther. 2023 Aug 22;25:152. doi: 10.1186/s13075-023-03109-4 (PMC10463648; doi:10.1186/s13075-023-03109-4)
Supplement: Supplementary file 2 — Additional file 2: Supplementary Table S2. PCR primers used in this study. [file 13075_2023_3109_MOESM2_ESM.docx]

**Supplementary Table S2. PCR primers used in this study**

| Gene Name | Forward primer (5'-3') | Reverse primer (5'-3') |
| --- | --- | --- |
| *GAPDH* | TCAACGACCACTTTGTCAAGCTCA | GCTGGTGGTCCAGGGGTCTTACT |
| *BCL6* | GCCGGCTGACAGCTGTATC | CGGAGACGATTAAGGTTGAGAA |
| *LEF1* | TGCCAAATATGAATAACGACCCA | GAGAAAAGTGCTCGTCACTGT |
| *MAF* | GGCATGCTAATCGCGTGAGA | GCTTCTCGTAGATGCCAGGA |
| *PRDM1* | AAGCAACTGGATGCGCTATGT | GGGATGGGCTTAATGGTGTAGAA |
| *TCF7* | CTGGCTTCTACTCCCTGACCT | ACCAGAACCTAGCATCAAGGA |
| *TOX2* | AGGCCAAGAACCCGAAGAAG | TTTGGACACGTCACCGAAAGT |
