# Supplementary material for: Iguratimod suppresses Tfh cell differentiation in primary Sjögren’s syndrome patients through inhibiting Akt/mTOR/STAT3 signaling
Source: Arthritis Res Ther. 2023 Aug 22;25:152. doi: 10.1186/s13075-023-03109-4 (PMC10463648; doi:10.1186/s13075-023-03109-4)
Supplement: Supplementary file 7 — Additional file 7: Supplementary Figure S1. Gating strategy of peripheral T cells and B cells. [file 13075_2023_3109_MOESM7_ESM.docx]

**Supplementary Figure S1.** Gating strategy of peripheral T cells and B cells.
