# Supplementary material for: Iguratimod suppresses Tfh cell differentiation in primary Sjögren’s syndrome patients through inhibiting Akt/mTOR/STAT3 signaling
Source: Arthritis Res Ther. 2023 Aug 22;25:152. doi: 10.1186/s13075-023-03109-4 (PMC10463648; doi:10.1186/s13075-023-03109-4)
Supplement: Supplementary file 8 — Additional file 8: Supplementary Figure S2. Peripheral T cell subsets in IGU-treated pSS patients. [file 13075_2023_3109_MOESM8_ESM.docx]

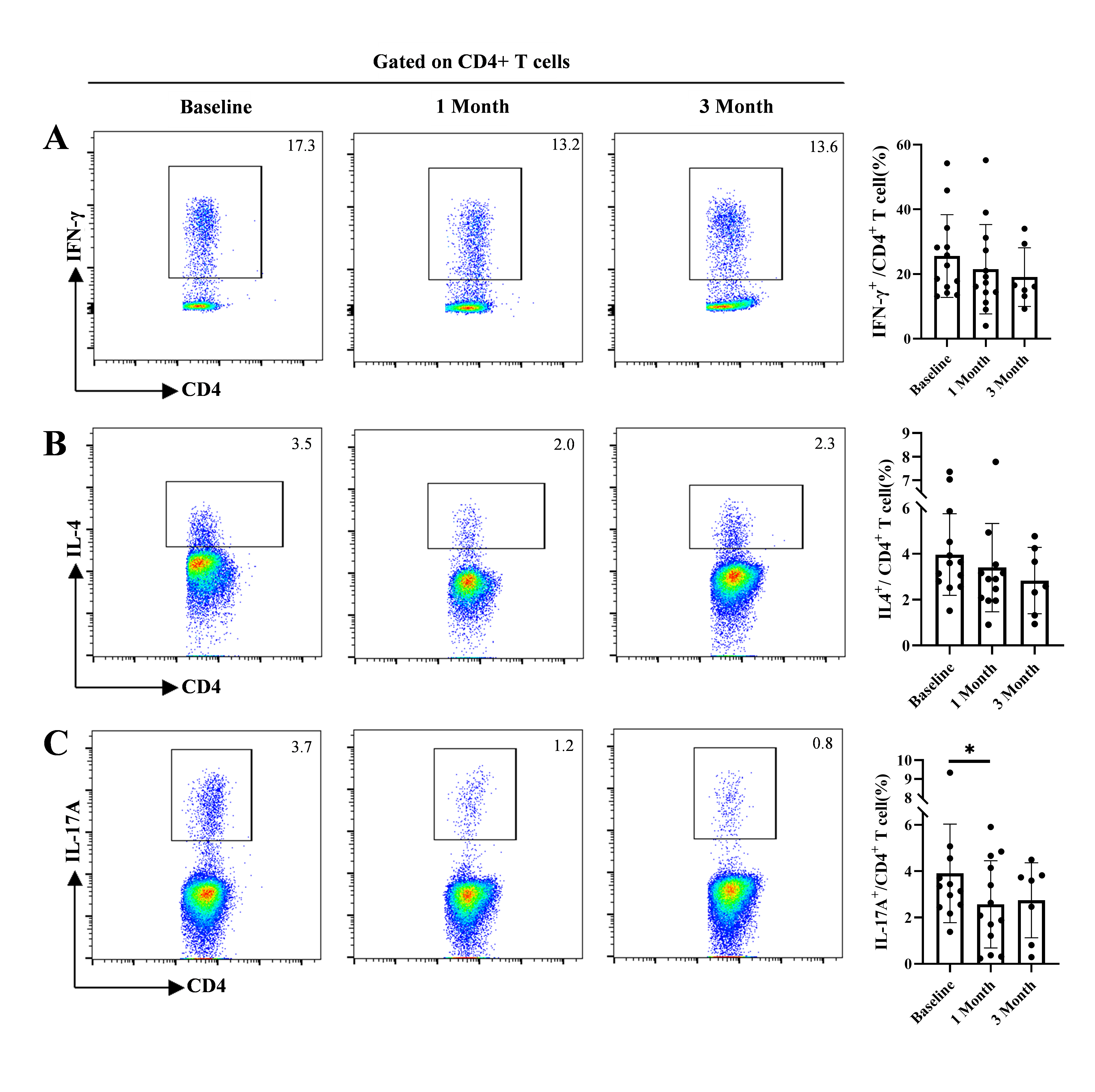


**Supplementary Figure S2.** Peripheral T cell subsets in IGU-treated pSS patients.

Flowcytometry analysis of (A) CD4^+^IFN-γ^+^ Th1, (B) CD4^+^IL-4^+^ Th2, (C) CD4^+^IL-17A^+^ Th17 cells isolated from peripheral blood of pSS patients (n=13). Data were presented as mean ± SD. Data were obtained from independent experiments. *p <0.05 by ANOVA.
