# Supplementary material for: Iguratimod suppresses Tfh cell differentiation in primary Sjögren’s syndrome patients through inhibiting Akt/mTOR/STAT3 signaling
Source: Arthritis Res Ther. 2023 Aug 22;25:152. doi: 10.1186/s13075-023-03109-4 (PMC10463648; doi:10.1186/s13075-023-03109-4)
Supplement: Supplementary file 9 — Additional file 9: Supplementary Figure S3. Clinical and laboratory improvement in IGU-treated pSS patients. [file 13075_2023_3109_MOESM9_ESM.docx]

**
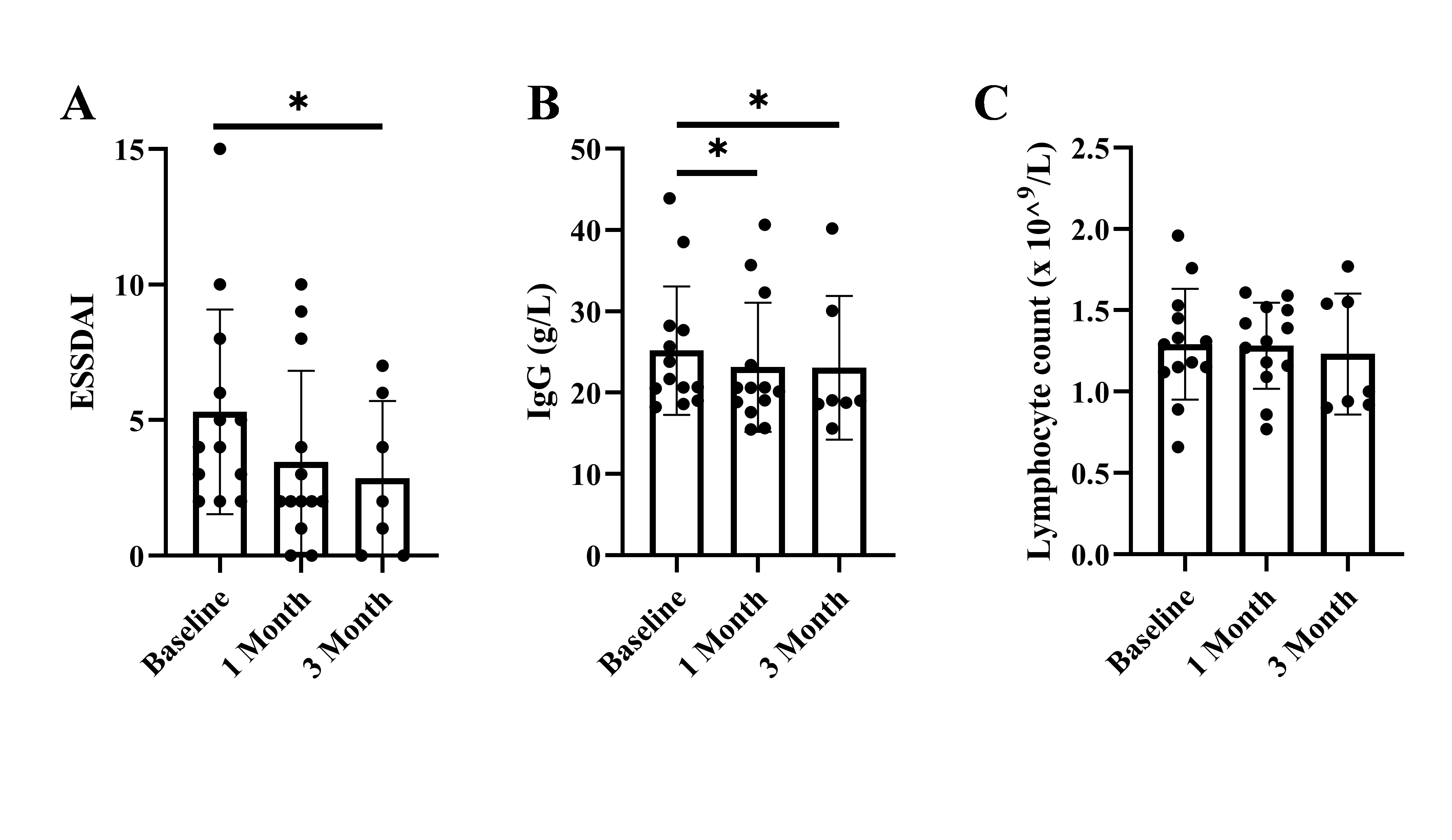
**

**Supplementary Figure S3.** Clinical and laboratory improvement in IGU-treated pSS patients.

Change of (A) ESSDAI, (B) IgG, and (C) lymphocyte counts in IGU-treated pSS patients. Data were presented as mean ± SD. *p <0.05 by ANOVA.
