# Supplementary material for: Iguratimod suppresses Tfh cell differentiation in primary Sjögren’s syndrome patients through inhibiting Akt/mTOR/STAT3 signaling
Source: Arthritis Res Ther. 2023 Aug 22;25:152. doi: 10.1186/s13075-023-03109-4 (PMC10463648; doi:10.1186/s13075-023-03109-4)
Supplement: Supplementary file 10 — Additional file 10: Supplementary Figure S4. Correlation analysis of Tfh cells with B cells and ESSDAI in IGU-treated pSS patients. [file 13075_2023_3109_MOESM10_ESM.docx]

**
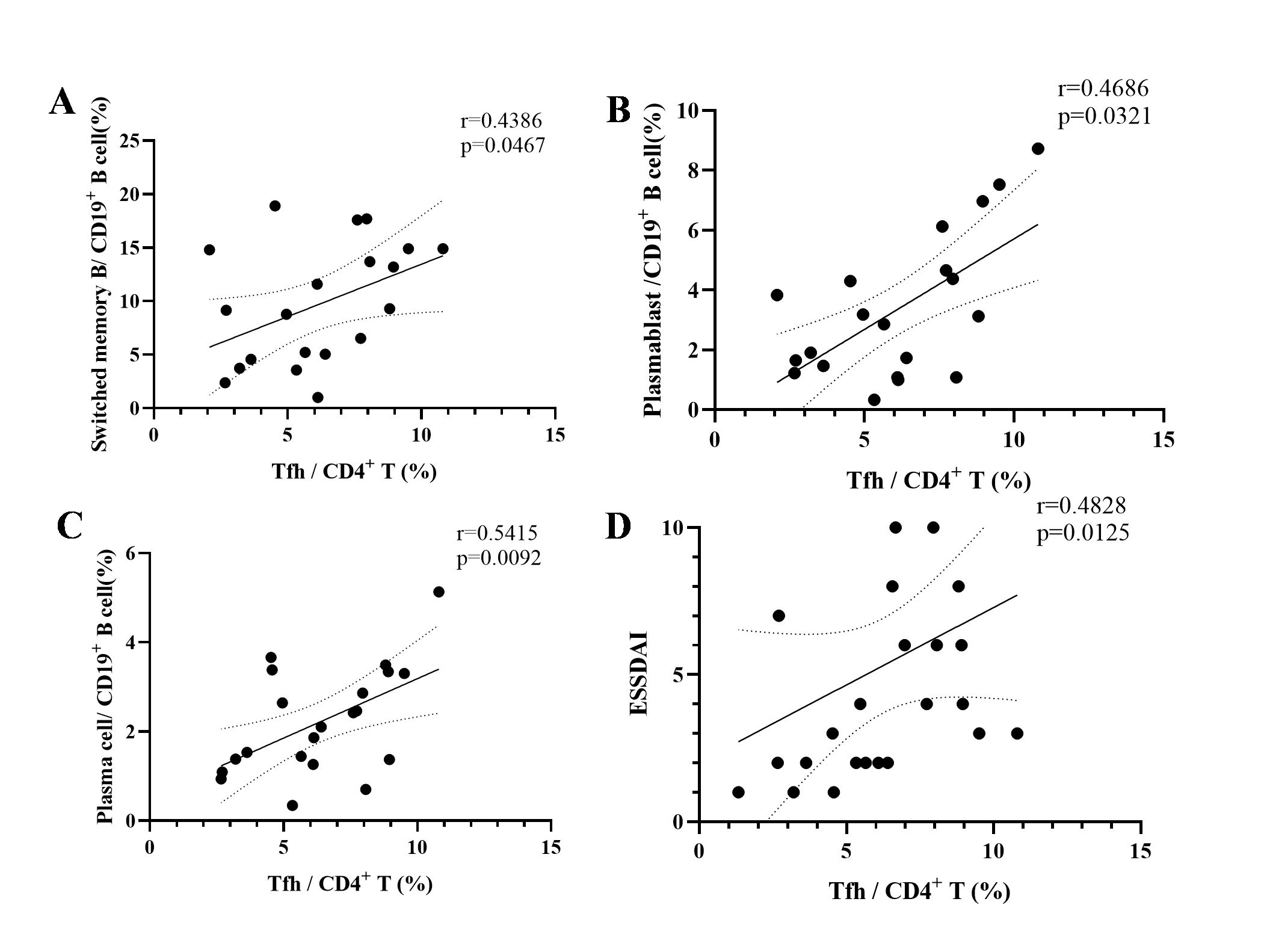
**

**Supplementary Figure S4.** Correlation analysis of Tfh cells with B cells and ESSDAI in IGU-treated pSS patients.

Correlation analysis of Tfh cells with (A) switched memory B cells, (B) plasmablasts, (C) plasma cells, and (D) ESSDAI in IGU-treated pSS patients.
