# Supplementary material for: Iguratimod suppresses Tfh cell differentiation in primary Sjögren’s syndrome patients through inhibiting Akt/mTOR/STAT3 signaling
Source: Arthritis Res Ther. 2023 Aug 22;25:152. doi: 10.1186/s13075-023-03109-4 (PMC10463648; doi:10.1186/s13075-023-03109-4)
Supplement: Supplementary file 12 — Additional file 12: Supplementary Figure S6. IGU inhibits T cell activation and proliferation. [file 13075_2023_3109_MOESM12_ESM.docx]

**

**

**Supplementary Figure S6.** IGU inhibits T cell activation and proliferation.

Flowcytometry analysis of (A) CD69 expression, (B) CD25 expression, (C) CFSE-diluted proliferation and (D) Annexin V 7-AAD apoptosis of naïve CD4^+^ T cells (n=5) stimulated with anti-CD3 and anti-CD28 for 3 days. Data were presented as mean ± SD. Data were obtained from two independent experiments. *p <0.05, **p <0.01 by ANOVA.
