# Supplementary material for: Iguratimod suppresses Tfh cell differentiation in primary Sjögren’s syndrome patients through inhibiting Akt/mTOR/STAT3 signaling
Source: Arthritis Res Ther. 2023 Aug 22;25:152. doi: 10.1186/s13075-023-03109-4 (PMC10463648; doi:10.1186/s13075-023-03109-4)
Supplement: Supplementary file 13 — Additional file 13: Supplementary Figure S7. IGU inhibits Th1, Th2 and Th17 cell differentiation. [file 13075_2023_3109_MOESM13_ESM.docx]

**

**

**Supplementary Figure S7.** IGU inhibits Th1, Th2 and Th17 cell differentiation.

Flow cytometry analysis of (A) CD4^+^IFN-γ^+^ Th1, (B) CD4^+^IL-4^+^ Th2 and (C) CD4^+^IL-17A^+^ Th17 cells differentiated from pSS naive CD4^+^ T cells (n=4) stimulated under Th1, Th2 or Th17 condition for 5 days, respectively. Data were presented as mean ± SD. Data were obtained from two independent experiments. *p <0.05, **p <0.01, ***p <0.001 by ANOVA.
