# Supplementary material for: Iguratimod suppresses Tfh cell differentiation in primary Sjögren’s syndrome patients through inhibiting Akt/mTOR/STAT3 signaling
Source: Arthritis Res Ther. 2023 Aug 22;25:152. doi: 10.1186/s13075-023-03109-4 (PMC10463648; doi:10.1186/s13075-023-03109-4)
Supplement: Supplementary file 14 — Additional file 14: Supplementary Figure S8. IGU inhibits healthy control Tfh cell differentiation. [file 13075_2023_3109_MOESM14_ESM.docx]

**

**

**Supplementary Figure S8.** IGU inhibits healthy control Tfh cell differentiation.

Flowcytometry analysis of (A) CD4^+^CXCR5^+^, (B) CD4^+^CXCR5^+^PD-1^+^, (C) CD4^+^CXCR5^+^ICOS^+^, and (D) CD4^+^IL-21^+^ Tfh cells differentiated from healthy control naive CD4^+^ T cells (n=6) stimulated under Tfh condition for 5 days. Data were presented as mean ± SD. Data were obtained from three independent experiments. *p <0.05, **p <0.01, ***p <0.001 by ANOVA.
