# Supplementary material for: Iguratimod suppresses Tfh cell differentiation in primary Sjögren’s syndrome patients through inhibiting Akt/mTOR/STAT3 signaling
Source: Arthritis Res Ther. 2023 Aug 22;25:152. doi: 10.1186/s13075-023-03109-4 (PMC10463648; doi:10.1186/s13075-023-03109-4)
Supplement: Supplementary file 15 — Additional file 15: Supplementary Figure S9. IGU suppresses B cell differentiation. [file 13075_2023_3109_MOESM15_ESM.docx]

**
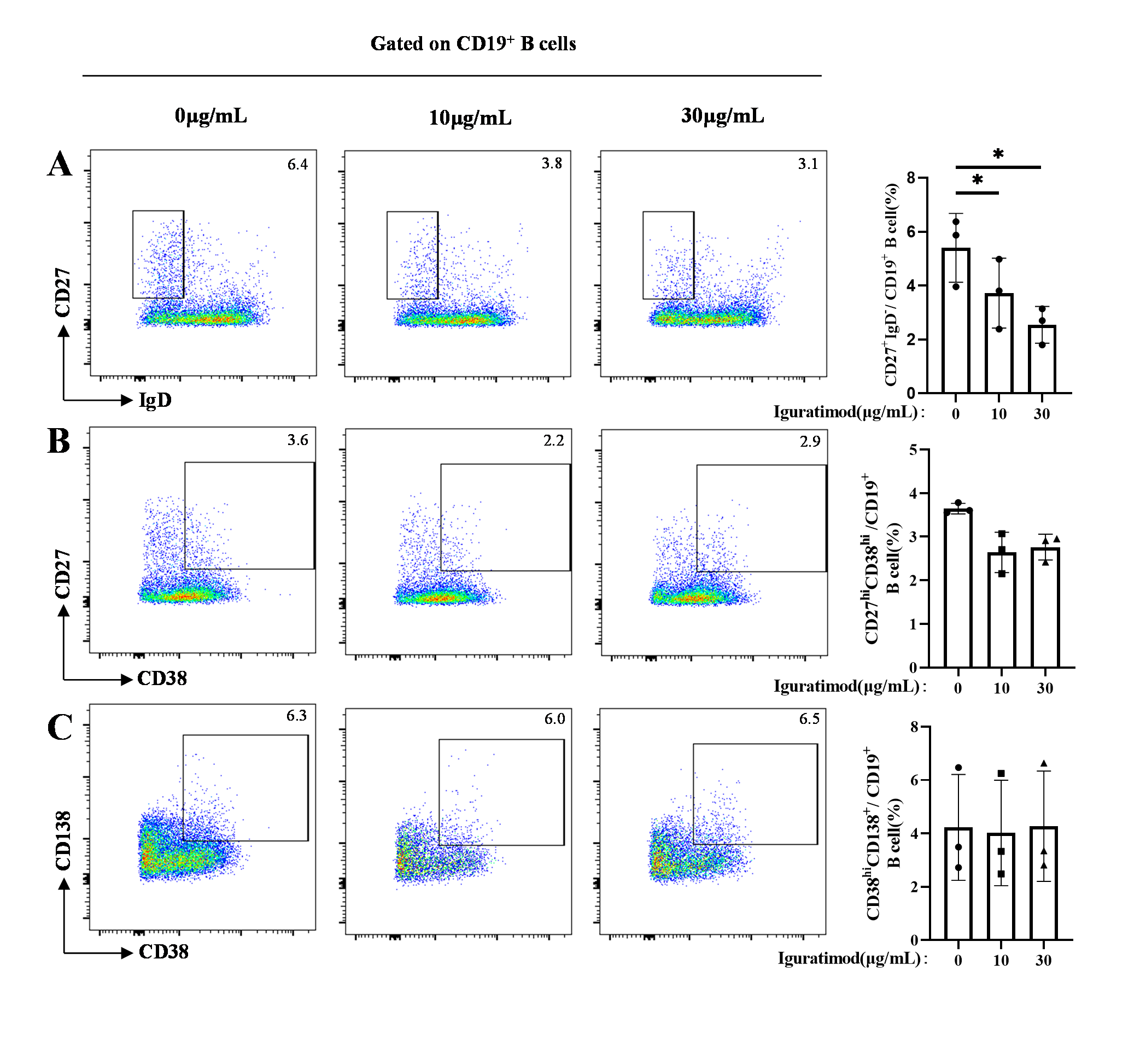
**

**Supplementary Figure S9**. IGU inhibits B cell differentiation.

Flowcytometry analysis of (A) CD27^+^IgD^-^ switched memory B cells, (B) CD27^hi^CD38^hi^ plasmablasts, and (C) C38^hi^CD138^+^ plasma cells differentiated from naïve B cells (n=3) treated with IGU or DMSO for 6 days. Data were presented as mean ± SD and were obtained from two independent experiments. *p <0.05 by ANOVA.
