# Supplementary material for: Iguratimod suppresses Tfh cell differentiation in primary Sjögren’s syndrome patients through inhibiting Akt/mTOR/STAT3 signaling
Source: Arthritis Res Ther. 2023 Aug 22;25:152. doi: 10.1186/s13075-023-03109-4 (PMC10463648; doi:10.1186/s13075-023-03109-4)
Supplement: Supplementary file 17 — Additional file 17: Supplementary Figure S11. Gene ontology analysis of IGU-treated CD4+ T cells. [file 13075_2023_3109_MOESM17_ESM.docx]

**
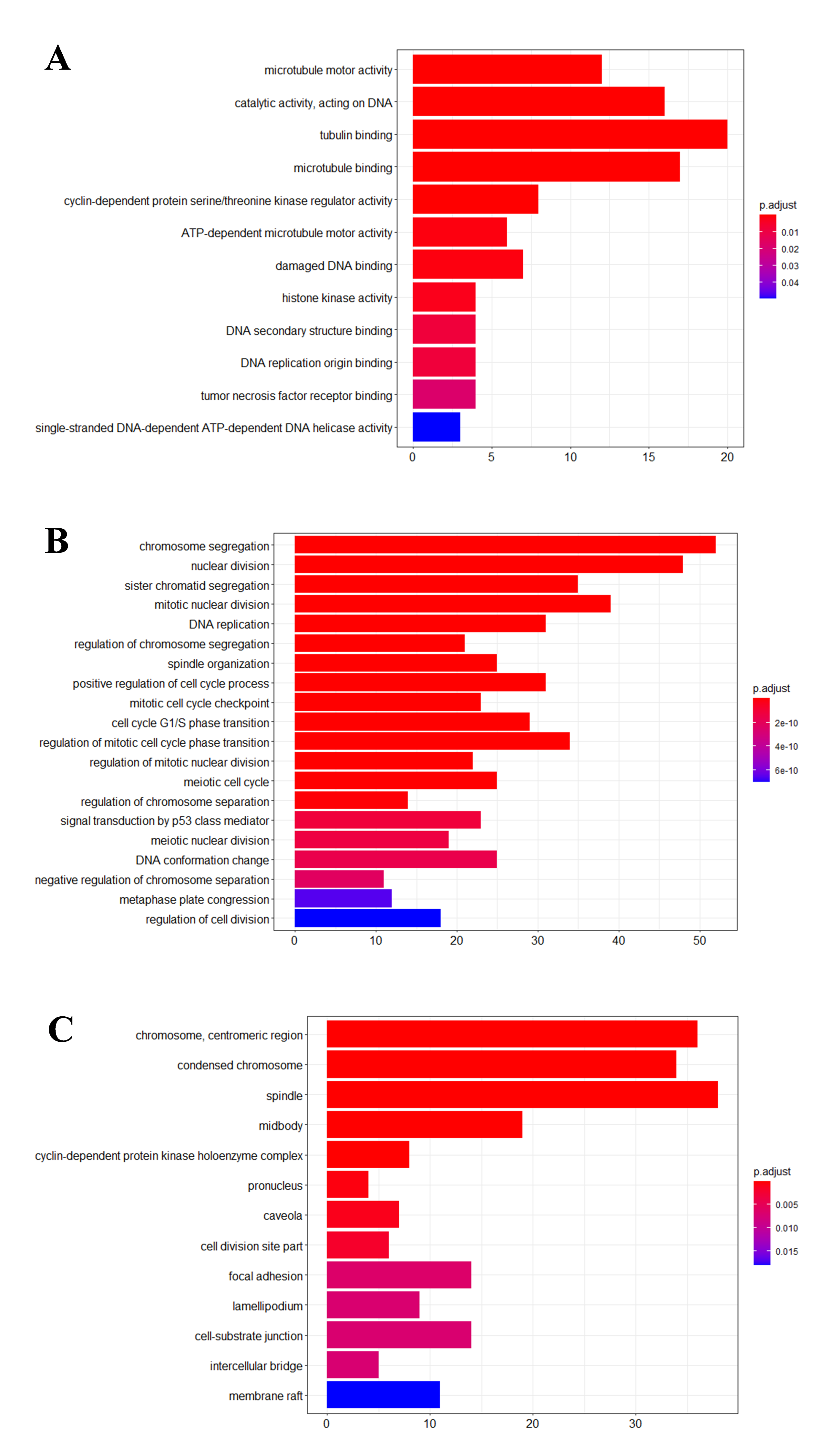
**

**Supplementary Figure S11.** Gene ontology analysis of IGU-treated CD4^+^ T cells.

Gene ontology analysis of (A) molecular function, (B) biological process, and (C) cellular component on DEGs between IGU -treated (n = 3) and untreated (n = 3) naïve CD4^+^ T cells under Tfh condition.
