# Supplementary material for: Iguratimod suppresses Tfh cell differentiation in primary Sjögren’s syndrome patients through inhibiting Akt/mTOR/STAT3 signaling
Source: Arthritis Res Ther. 2023 Aug 22;25:152. doi: 10.1186/s13075-023-03109-4 (PMC10463648; doi:10.1186/s13075-023-03109-4)
Supplement: Supplementary file 18 — Additional file 18: Supplementary Figure S12. AKT activator restored IGU-suppressed mTOR and STAT3 phosphorylation and T cell proliferation [file 13075_2023_3109_MOESM18_ESM.docx]

**
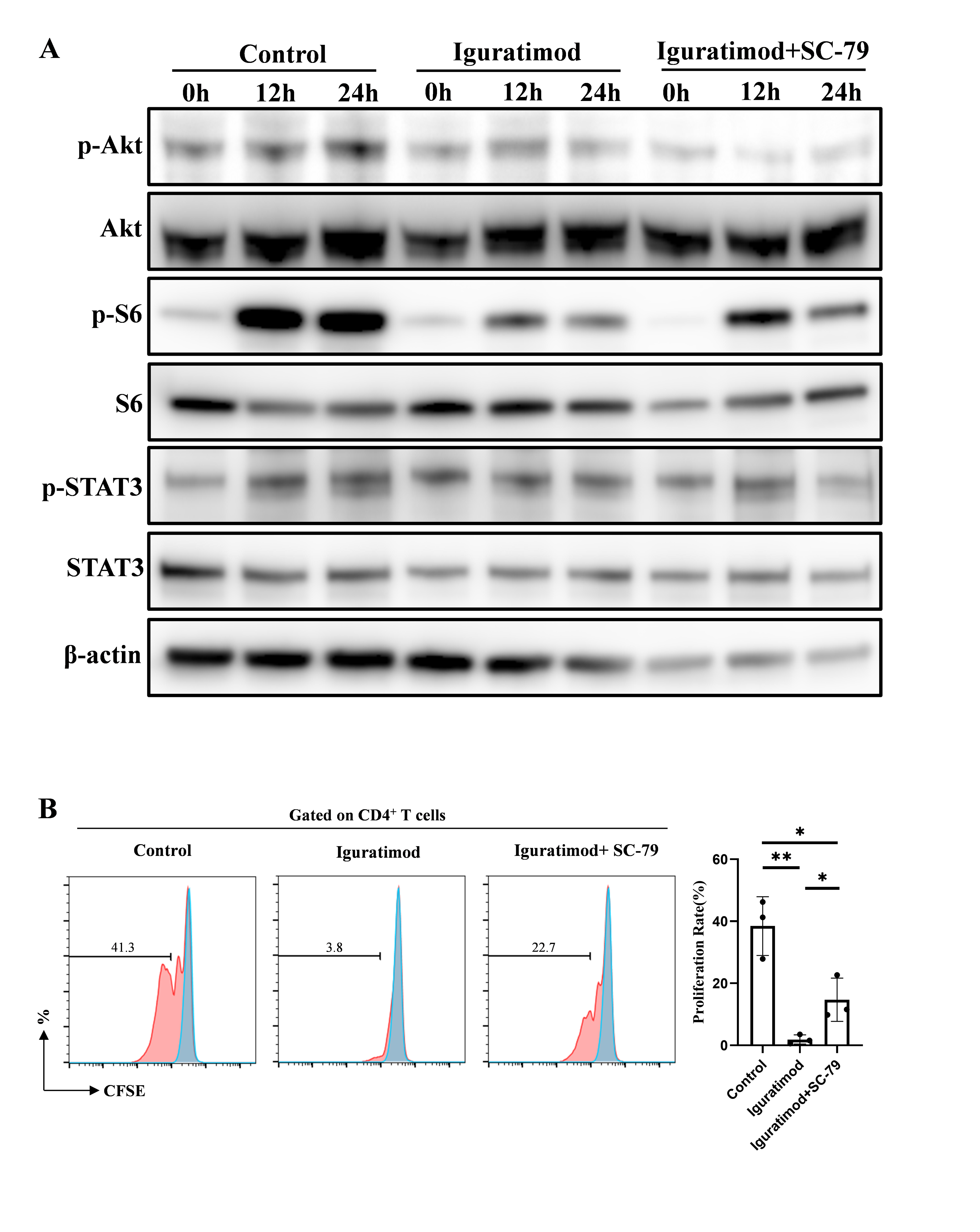
**

**Supplementary Figure S12**. AKT activator restored IGU-suppressed mTOR and STAT3 phosphorylation, and T cell proliferation.

(A) Western blot analysis of Akt, S6 and STAT3 in DMSO- (Control), IGU- (Iguratimod) or IGU and SC-79-treated (Iguratimod+SC-79) naïve CD4^+^ T cells incubated under Tfh condition for 0, 12, or 24 hours. (B) Flowcytometry analysis of CFSE-diluted proliferation of naïve CD4^+^ T cells (n=3) stimulated with anti-CD3 and anti-CD28 under Tfh condition for 3 days. Data were presented as mean ± SD and were obtained from two independent experiments. *p <0.05, **p <0.01 by ANOVA.
