# Supplementary material for: Iguratimod suppresses Tfh cell differentiation in primary Sjögren’s syndrome patients through inhibiting Akt/mTOR/STAT3 signaling
Source: Arthritis Res Ther. 2023 Aug 22;25:152. doi: 10.1186/s13075-023-03109-4 (PMC10463648; doi:10.1186/s13075-023-03109-4)
Supplement: Supplementary file 19 — Additional file 19: Supplementary Figure S13. Diagram of mechanism of IGU-inhibited Tfh cell differentiation [file 13075_2023_3109_MOESM19_ESM.docx]

**

**

**Supplementary Figure S13.** Diagram of mechanism of IGU-inhibited Tfh cell differentiation.

IGU binds PDK1 and inhibits phosphating Akt, subsequently attenuates mTORC1 activation and downstream STAT3 phosphorylation, which acts as transcription factor and promotes *BCL6* and inhibits *PRDM1* transcription. Therefore, IGU inhibits Tfh cell differentiation through upregulating *PRDM1* and downregulating *BCL6* through interacting with PDK1 to inhibit Akt-mTORC1-STAT3 pathway. *BCL6*: B-cell lymphoma. mTORC1: mammalian target of rapamycin complex 1. PDK1: Pyruvate dehydrogenase kinase 1. *PRDM1*: PR domain zinc finger protein 1. STAT3, signal transducer and activator of transcription 3.
